# Happily Distracted: Mood and a Benefit of Attention Dysregulation in Older Adults

**DOI:** 10.3389/fpsyg.2012.00399

**Published:** 2012-10-16

**Authors:** Renée K. Biss, Jennifer C. Weeks, Lynn Hasher

**Affiliations:** ^1^Department of Psychology, University of TorontoToronto, ON, Canada; ^2^Rotman Research Institute of Baycrest CentreToronto, ON, Canada

**Keywords:** aging, positive affect, mood, attention regulation, distraction, inhibition

## Abstract

Positive mood states are believed to broaden the focus of attention in younger adults, but it is unclear whether the same is true for older adults. Here we examined one consequence of broader attention that has been shown in young adults: that memory for distraction is greater for those in a positive mood. In the current study, positive and neutral moods were induced in older adults (*M* = 67.9) prior to a 1-back task in which participants were instructed to attend to relevant pictures and ignore distracting words. Following a 10-min filled interval, participants performed a word fragment completion task that tested implicit memory for the distracting words from the 1-back task. Older adults in the positive mood group showed greater implicit memory for previous distraction compared to those in the neutral mood group. These findings suggest that affect influences the ability to regulate attention in a similar manner for younger and older adults.

## Introduction

Affective states are believed to serve a variety of functions including regulating the bandwidth of attention. Relative to negative states, positive affective states broaden both spatial (Schmitz et al., 2009) and temporal attention (Vermeulen, 2010), possibly because positive affect is a signal of relative safety to explore the environment (Fredrickson, 1998; Friedman and Förster, 2010).

However, attentional broadening can also have negative effects on cognition which can manifest in a number of ways, including increased susceptibility to distraction. For example, positive affect can increase interference from distractors during both flanker (Rowe et al., 2007) and set switching tasks (Dreisbach and Goschke, 2004) relative to neutral or negative affect. As well, positive affect decreases performance on tasks requiring inhibitory control, such as directed forgetting (Bäuml and Kuhbandner, 2009) and negative affective priming (Goeleven et al., 2007). Therefore, it seems plausible that positive affect may impair the ability to ignore irrelevant distraction by slackening inhibitory control.

Given the apparently increased attention paid to distractors as a result of positive mood, it is also possible that positive affect can actually enhance performance should previously irrelevant items later become relevant. Previous research demonstrated that younger adults in both naturally occurring (Biss et al., 2010) and experimentally induced positive moods (Biss and Hasher, 2011) showed greater implicit use of previous distraction compared to those in more neutral moods. Older adults, a population considered to have reduced inhibitory control abilities relative to younger adults (Hasher et al., 1999), also show greater implicit memory for distraction (Rowe et al., 2006).

An open question is whether the tendency for positive affect to broaden attention and increase the implicit use of distraction also extends to older adults who generally show less control over attention than their younger counterparts (Rowe et al., 2006).

In the current study, we tested whether mood influences older adults’ ability to ignore distraction in the same manner as we have demonstrated in younger adults. We used a paradigm that has previously been used to demonstrate mood differences in implicit memory for distraction among younger adults (Biss and Hasher, 2011) as well as age differences in priming for distraction (Rowe et al., 2006). Positive and neutral affect were induced in older adults prior to a 1-back task in which participants responded to relevant pictures and were instructed to ignore superimposed irrelevant words. Following a 10-min filled delay, we tested implicit knowledge of the previously distracting words using a fragment completion task. If positive affect increases susceptibility to distraction in older adults as it appears to in younger adults, then older adults in an induced positive affect group should show enhanced priming for previous distraction compared to those in a neutral affect group.

## Materials and Methods

### Participants

Sixty older adult participants (*M* = 67.9, SD = 4.6) were recruited from the community and paid for their participation. Participants were randomly assigned to either the positive (*n* = 30) or neutral (*n* = 30) mood induction condition. All participants were free of neurological or psychiatric illness, and were native English speakers or had learned English by age 6. In keeping with the goal of assessing implicit memory for distraction, we replaced data from participants who reported being aware of the connection between the 1-back and fragment completion tasks (three in the neutral condition). In addition, we replaced participants who appeared to focus directly on the distraction, either by being very slow (more than 2.5 SDs slower than the group mean; one participant in the positive condition) or very inaccurate on the 1-back task (50% accuracy or below; three each in the positive and neutral conditions) relative to the majority of participants tested here and in prior work (Rowe et al., 2006; Biss and Hasher, 2011). The remaining participants had an average of 17.1 years of education (SD = 4.4), and scored 35.3 (SD = 3.5) on the Shipley (1946) vocabulary test. Mood groups did not differ based on age, education, or vocabulary scores, *t*s < 1. The experimental procedures were approved by the Social Sciences, Humanities, and Education Research Ethics Board at the University of Toronto, and informed consent was obtained from all participants prior to the experiment.

### Materials

#### Mood induction

One hundred forty-six pictures were selected from the International Affective Picture System (IAPS; Lang et al., 2008) based on the norms provided in the IAPS database. Pictures selected for the neutral condition had valence ratings between 4.5 and 5.5 (*M* = 5.1, SD = 0.3), while pictures selected for the positive affect induction had valence ratings of seven or higher (*M* = 7.5, SD = 0.3). Arousal ratings did not differ between neutral (*M* = 4.1, SD = 0.9) and positive pictures (*M* = 4.2, SD = 0.5), *t*(144) < 1. Although these IAPS norms include ratings from younger adults only, other normative data suggests that older adults’ valence ratings of IAPS pictures are strongly associated with normative ratings collected in younger adults (Grühn and Scheibe, 2008). Sound clips free of verbal material were also selected for use in the mood induction. In the neutral affect condition, a sound clip with ambient street sounds was played. In the positive affect condition, participants heard a jazzy version of Bach’s *Brandenberg Concerto No. 3* that has previously been used to induce positive mood in young adults (e.g., Rowe et al., 2007; Biss and Hasher, 2011).

#### Mood ratings

Participants rated the pleasantness of their moods on a nine-point scale adapted from Rowe et al. (2007), ranging from 1 (*not at all pleasant*) to 9 (*extremely pleasant*). They also rated arousal on a nine-point scale that ranged from 1 (*very calm*) to 9 (*very aroused*).

#### 1-back task pictures

Fifty-five nameable line drawings were selected from Snodgrass and Vanderwart (1980) and colored red. Fifty of these pictures were superimposed with distraction: 30 with non-words, 10 with critical primed words, and 10 with filler words, which were used to reduce awareness of the connection between tasks.

#### Word stimuli and fragments

Two lists of 10 words and their corresponding fragments were chosen, and were matched for the number of letters in both the provided fragment and critical solution word, as well as based on previously collected baseline completion rates (Ikier, 2005). Lists were counterbalanced such that each participant was exposed to one list as critical primed words in the 1-back task, and solved fragments from both lists in the fragment completion task. Completion from the control list not seen in the 1-back task was used to calculate baseline completion for critical words. The critical words were between five and eight letters long (*M* = 6.0, SD = 1.1), and fragments contained between two and five letters (*M* = 3.4, SD = 0.8). All fragments had multiple solutions (e.g., B _ T T _ _ S could be solved using BUTTERS, BATTLES, or BUTTONS), only one of which (e.g., BUTTONS) was presented during the course of the experiment. Ten easy filler fragments also appeared in the fragment completion task in order to limit participants’ awareness of the connection between tasks and to ensure that they felt successful about their fragment completion performance.

### Procedure

Before the experimental tasks began, participants rated their mood pleasantness and arousal. A 6-min mood induction procedure followed, during which participants viewed the IAPS pictures and listened to the corresponding sound clip using headphones. Each picture appeared on the computer screen for 5 s. Participants were instructed to relax and think about how the pictures and sounds made them feel. Participants rated their mood pleasantness and arousal again after the mood induction in order to determine its effectiveness.

The 1-back task followed. Participants were instructed to attend to a series of line drawings presented individually on the computer screen, pressing a key whenever consecutive pictures were identical, and to ignore the words and non-words that were superimposed over the pictures. Each overlapping picture and word/non-word appeared for 1000 ms, with an ISI of 500 ms. There were 10 pictures that repeated, requiring a key press; these repeated pictures occurred randomly amid novel pictures, with lags of two to six pictures in between repeated picture trials. Picture and word stimuli were assigned randomly to repeated trials, and no words were repeated during the task. Responses on these trials were recorded to calculate accuracy and RT. The 55 total trials proceeded in the following sequence: five pictures presented alone, eight pictures with superimposed non-words, 34 pictures superimposed with either non-words, filler words, or primed words, and eight pictures with superimposed non-words.

Participants then performed a non-verbal filler task for 10 min. Following the filler task, participants rated their mood pleasantness and arousal.

During the fragment completion task, participants viewed 30 word fragments, each presented individually on the computer screen for 3000 ms, with an ISI of 500 ms. They were instructed to respond out loud with the first word that came to mind, and the experimenter recorded their responses. The fragments included 10 easy filler fragments, 10 primed items presented as distraction during the 1-back task, and 10 control items from the list not seen in the 1-back task.

Following the fragment completion task, participants rated their mood pleasantness and arousal a final time. Participants were then given an awareness questionnaire to ensure that the test was implicit for all participants: they were asked if they had noticed a connection between any of the experimental tasks, and if so, to describe the connection. Finally, participants completed a background questionnaire and were debriefed. All participants watched a brief comedic video clip before leaving as a mood reinstatement.

## Results

**Figure 1:**
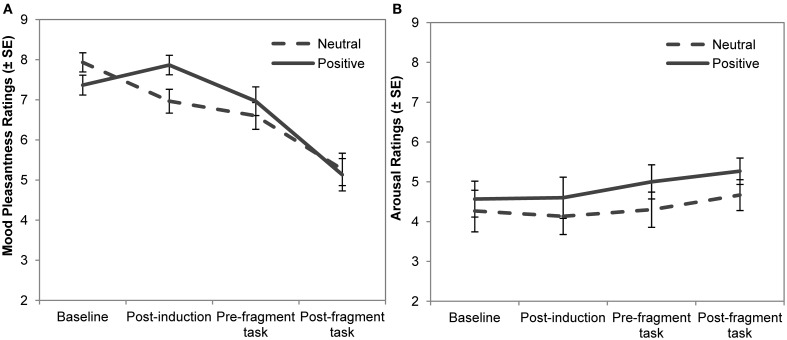
**Ratings made at various points during the experiment for (A) mood pleasantness, and (B) arousal**. Error bars indicate standard error of the mean.

Mood pleasantness and arousal ratings made during the experiment are reported in Figure 1. Mood pleasantness ratings were entered into an ANOVA with mood group (neutral, positive) as a between-subject factor and rating time (baseline, post-induction, pre-fragment, post-fragment) as a within-subject factor. Greenhouse–Geisser corrections for degrees of freedom were used whenever appropriate. Both the absence of a main effect for mood, *F* < 1, and the presence of an effect of rating time, *F*(2.3,134) = 48.35, *p* < 0.001, were qualified by an interaction between mood group and rating time, *F*(2.3, 134) = 3.98, *p* = 0.02, η_p_^2^ = 0.06. Planned comparisons showed that participants in the positive mood condition had higher pleasantness ratings following the mood induction, *t*(58) = 2.34, *p* = 0.02, *d* = 0.61, but not at any other point during the experiment, *p*s > 0.10. Arousal ratings were also entered into a mood group x rating time ANOVA. Neither of the main effects nor the interaction was significant, *p*s > 0.22.

There was no difference in accuracy on the 1-back task for participants in the neutral (*M* = 91%, SD = 12%) and positive affect (*M* = 90%, SD = 11%) groups, *t*(58) < 1. Similarly, there was no difference in 1-back task RTs between the neutral (*M* = 599 ms, SD = 128) and positive (*M* = 619 ms, SD = 111) groups, *t*(58) < 1.

Performance on the word fragment completion task is shown in Table 1. Affect groups did not differ in completion of filler or control fragments, *t*s < 1. Priming scores were calculated using group baseline data, by taking each individual’s primed fragment completion and subtracting average control fragment completion for their affect group. Consistent with our prediction, priming for distraction was greater among older adults in the positive affect compared to the neutral affect condition, *t*(58) = 1.99, *p* = 0.05, *d* = 0.51.

**Table 1:** **Mean percentage completion (SD) on the fragment completion task**.

Measure	Affect group	
	Positive	Neutral
Filler fragment completion	71 (18)	67 (17)
Primed fragment completion	19.3 (13)	15.7 (10)
Control fragment completion	8.3 (11)	10.7 (9)
Priming	11.0 (13)	5.0 (11)

To further investigate how mood influenced priming for distraction, we analyzed whether there was an association between mood ratings and fragment completion performance when baseline mood was controlled. Across all participants, there was a significant partial correlation between post-induction pleasantness ratings and priming for distraction, pr = 0.33, p = 0.01. Post-induction pleasantness ratings were not associated more generally with fragment completion performance, as indexed by partial correlations with control fragment completion, pr = 0.15, *p* = 0.25 or with filler fragment completion, pr = 0.20, *p* = 0.14. Mood at retrieval was not associated with priming: There was no partial correlation between pleasantness ratings made prior to the fragment completion task and priming for distraction, pr = 0.18, *p* = 0.16.

## Discussion

Older adults in an induced positive mood showed greater priming for distraction compared to age mates in a neutral mood condition. Although the two mood groups did not differ in 1-back task performance, they did differ in subsequent knowledge for distraction presented during that task. In addition, mood pleasantness ratings made prior to the 1-back task were associated with the extent of priming when baseline mood was controlled. Ratings made at other times did not differ between mood groups, and had no influence on priming for distraction. Thus, the effect of mood was specific to attention at encoding, and in particular, there was no evidence that mood at retrieval mattered. Our results suggest that positive mood in older adults results in increased encoding of information that was never relevant to a task.

These findings support the idea that positive affect relaxes attentional control processes that otherwise restrict goal-irrelevant information from reaching the focus of attention (Hasher et al., 1999), an effect that is generally considered an impairing effect of emotion on attentional selectivity (e.g., Dreisbach and Goschke, 2004; Goeleven et al., 2007; Rowe et al., 2007). Here, we have shown that reduced inhibitory control and broadening of attention under positive affect (Fredrickson, 2001) can also have an enhancing effect. This enhancement was seen here on an implicit memory task which tested for tacit knowledge of previous distraction. Given the similarity of these results to those shown by younger adults (Biss and Hasher, 2011), positive affect appears to have a similar effect on the downstream benefits of distractibility among both younger and older adults.

Taken together with evidence that aging is associated with increased positive affect (e.g., Mroczek and Kolarz, 1998; Stone et al., 2010; Carstensen et al., 2011; Reed and Carstensen, 2012), our results suggest that age differences in affect may contribute to at least some age differences in cognition. Older adults generally have difficulty down-regulating the processing of irrelevant information, and show greater implicit knowledge about previous distraction compared to younger adults (Rowe et al., 2006). Given the evidence here that positive affect modulates older adults’ processing of distraction, greater positive affect among older adults may partially contribute to this age difference in attentional control. There is evidence that positive affect also influences the magnitude of age differences for other attention processes: Positive affect mediates the extent of age differences in alerting efficiency (Noh et al., 2012). Affective state may also contribute to age differences on the Tower of London task, a measure of executive function (Phillips et al., 2002). Phillips and colleagues found that, under induced neutral mood, there were no age differences in the ability to plan moves on the task. In contrast, positive mood decreased both younger and older adults’ planning ability, with older adults particularly disrupted relative to younger adults. This work highlights the importance of considering emotional and motivation influences on cognitive performance when studying age differences in cognition (see also Biss and Hasher, 2011; Hess et al., 2012 for a similar perspective).

Positive affective shifts associated with age may operate in parallel with normative changes in the structure and function of the brain (see Grady, 2008), potentially amplifying the cognitive consequences of these neural changes. Positive affect is thought to influence recruitment of prefrontal cortex and cingulate regions associated with executive control (Mitchell and Phillips, 2007), possibly via increased dopamine levels in these regions (Ashby et al., 1999). In particular, positive affect may influence the interaction between ventral emotional processing regions involved in automatic processing of salient information and dorsal control regions that downregulate processing of goal-irrelevant information from the environment (Drevets and Raichle, 1998; Dolcos and McCarthy, 2006). There is mounting evidence in favor of a functional dissociation between the dorsal executive control network and ventral emotion processing regions (see Dolcos et al., 2011). Dolcos and McCarthy (2006) found that poor working memory performance in the face of emotional distraction was associated with increased activity in ventral regions and decreased activity in dorsal regions, suggesting a trade-off between emotional and executive processes. Given recent fMRI evidence that younger and older adults recruit ventral affective areas during viewing of positive stimuli and dorsal executive control regions during viewing of negative stimuli (Ebner et al., 2012), it seems possible that positive affect’s impairing effect on attention is related to decreased blood flow to executive regions and increased blood flow to affective processing regions.

Prefrontal control regions have been implicated in age differences in susceptibility to distraction (e.g., Chao and Knight, 1997; Jonides et al., 2000; Gazzaley et al., 2008). While performing a 1-back task similar to that used here, older adults showed less activation in a frontoparietal control network than younger adults when instructed to ignore irrelevant words, and decreased activity in this control network was associated with greater priming for the irrelevant words later on (Campbell et al., 2012). Thus, positive emotion’s impairing effect on attention selectivity and enhancing effect on tacit knowledge of past distraction may amplify the effects of normal aging. Positive affect may act via ventral emotion processing regions to modulate older adults’ recruitment of prefrontal control regions that are already associated with age-related reductions, thus facilitating automatic or implicit processing of distraction. Ventral areas of the prefrontal cortex are likely to play a larger role in this process than are basic emotion areas such as the amygdala, considering recent event-related potential evidence suggesting that older adults’ visual attention is not modulated by the amygdala as is seen in younger adults (Pollock et al., 2012).

In a general sense, both positive affect and aging may result in a cognitive shift toward a more diffuse mental state that involves disengagement of executive control and greater reliance on more automatic processes. While there may be substantial cognitive costs, such as greater distractibility, there likely are also tangible benefits to this broadened mode of processing, including enhanced tacit knowledge of past distraction, as we have shown here.

## Conflict of Interest Statement

The authors declare that the research was conducted in the absence of any commercial or financial relationships that could be construed as a potential conflict of interest.
